# 558 Nurse-driven fluid resuscitation protocol - a quality improvement initiative

**DOI:** 10.1093/jbcr/irac012.186

**Published:** 2022-03-23

**Authors:** Kim Linticum, Shawn P Fagan, Bounthavy F Homsombath, Lynn Dowling, Mandy Rhodes

**Affiliations:** Joseph M. Still Burn Center at Doctors Hospital, Augusta, Georgia; Burn & Reconstructive Centers of America, Augusta, Georgia; Joseph M. Still Burn Center at Doctors Hospital Augusta, Augusta, Georgia; Joseph M. Still Burn Center at Doctors Hospital, Augusta, Georgia; Joseph M. Still Burn Center at Doctors Hospital, Augusta, Georgia

## Abstract

**Introduction:**

The migration of paper medical records to electronic health record (EHR) has been in process for a few years now and most facilities have achieved this successfully. EHR has streamlined care and documentation. Further developments such as Computerized Physician Order Entry (CPOE) has been noted as one of the most promising functionalities of Health Information Technology (HIT), as it allows providers to enter orders, medications, diagnostic tests, and procedures, with the intent of improving the clarity and specificity of physician orders, facilitating the rapid communication of orders to pharmacies, and providing significantly enhanced decision support capabilities compared to traditional handwritten orders.^1^

In our experience, initiation of CPOE has been beneficial in many ways, namely in decreasing medication errors related to handwritten orders. However, in the clinical scenario of acute resuscitation of a critically injured burn patient, the CPOE structure did not address each and every need that would arise, especially if fluid titration was necessary. Nursing staff were left unsure of what to do in terms of their role in adjusting fluids and assessing for adequacy of resuscitation. This led to gaps in care in which potential critical situations needed to be addressed. For instance, the possible development of abdominal compartment syndrome and how to respond to it was not part of CPOE set that was implemented. This was placing the patient at risk by delaying initiation of hemodynamic monitoring and delaying electrolyte replacement as well.

The goal of this study is to report the outcomes of this quality improvement initiative and to describe the resultant research that is in place to evaluate its effectiveness.

**Methods:**

This is a QI project that will identify and describe how a protocol was developed post- CPOE implementation to address gaps in nursing care during fluid resuscitation of critically ill burned patients

**Results:**

We created a protocol that allows the nurse to have better insight into what is happening with the patient and what physician orders are most pertinent at any particular time. The protocol sets parameters that alerts the nurse when additional intervention is necessary. For instance, monitoring for abdominal compartment syndrome begins once resuscitation exceeds 6 mL/kg/TBSA and allowing for the nurse to call the primary physician for hypotension that is refractory to fluid bolus. This was not clear before and nurses were not intervening appropriately, which resulted in gaps in delivery of care. We have not had to report any adverse or sentinel event related to fluid resuscitation since the implementation of this protocol.

**Conclusions:**

A nurse-driven protocol helped address gaps in care for nurses at the bedside during fluid resuscitation

